# Data Collection Mechanisms in Health and Wellness Apps: Review and Analysis

**DOI:** 10.2196/30468

**Published:** 2022-03-09

**Authors:** Ben Joseph Philip, Mohamed Abdelrazek, Alessio Bonti, Scott Barnett, John Grundy

**Affiliations:** 1 Deakin University Melbourne Australia; 2 School of Information Technology Faculty of Science, Engineering and Built Environment Deakin University Melbourne Australia; 3 Applied Artificial Intelligence Institute Deakin University Melbourne Australia; 4 Monash University Melbourne Australia

**Keywords:** data collection, mHealth apps, app review, app analysis, mHealth, mobile apps, development, data sharing, user experience, usability, automation, data reliability, mobile phone

## Abstract

**Background:**

There has been a steady rise in the availability of health wearables and built-in smartphone sensors that can be used to collect health data reliably and conveniently from end users. Given the feature overlaps and user tendency to use several apps, these are important factors impacting user experience. However, there is limited work on analyzing the data collection aspect of mobile health (mHealth) apps.

**Objective:**

This study aims to analyze what data mHealth apps across different categories usually collect from end users and how these data are collected. This information is important to guide the development of a common data model from current widely adopted apps. This will also inform what built-in sensors and wearables, a comprehensive mHealth platform should support.

**Methods:**

In our empirical investigation of mHealth apps, we identified app categories listed in a curated mHealth app library, which was then used to explore the Google Play Store for health and medical apps that were then filtered using our selection criteria. We downloaded these apps from a mirror site hosting Android apps and analyzed them using a script that we developed around the popular AndroGuard tool. We analyzed the use of Bluetooth peripherals and built-in sensors to understand how a given app collects health data.

**Results:**

We retrieved 3251 apps meeting our criteria, and our analysis showed that 10.74% (349/3251) of these apps requested Bluetooth access. We found that 50.9% (259/509) of the Bluetooth service universally unique identifiers to be known in these apps, with the remainder being vendor specific. The most common health-related Bluetooth Low Energy services using known universally unique identifiers were Heart Rate, Glucose, and Body Composition. App permissions showed the most used device module or sensor to be the camera (669/3251, 20.57%), closely followed by location (598/3251, 18.39%), with the highest occurrence in the *staying healthy* app category.

**Conclusions:**

We found that not many health apps used built-in sensors or peripherals for collecting health data. The small number of the apps using Bluetooth, with an even smaller number of apps using standard Bluetooth Low Energy services, indicates a wider use of proprietary algorithms and custom services, which restrict the device use. The use of standard profiles could open this ecosystem further and could provide end users more options for apps. The relatively small proportion of apps using built-in sensors along with a high reliance on manual data entry suggests the need for more research into using sensors for data collection in health and fitness apps, which may be more desirable and improve end user experience.

## Introduction

### Background

Mobile health (mHealth) apps support health delivery by the use of mobile devices such as mobile phones, wearables, and other wireless devices [1]. Several mHealth systems have been created for various apps, such as drug dosage reference [2,3], weight management [4], and monitoring cardiac health using wearable devices [5]. These mobile apps collect or generate health insights from three sources: external devices (Bluetooth or Wi-Fi-based sensors), built-in smartphone sensors, and manual data entry.

By 2017, it was estimated that more than 300,000 health apps were available in app stores, with a market growth of 25% each year [6,7]. The use of mobile apps along with wearables and external sensors has enabled self-monitoring of one’s health. They unobtrusively collect physiological data to provide better health outcomes and can also play an important role for patients living in remote areas with limited access to health care [4]. mHealth apps have been classified either as active or passive [8]—the former generate or derive health data using sensors, whereas the latter rely on manual user input.

The mHealth domain has seen a steady rise in smart wearable and fixed devices [9] that can be used to gather more detailed and accurate insights into people’s health [10]. According to Forbes, by 2022, their demand is expected to grow annually by approximately 20% [11]. This introduction of sensors has also opened up new avenues for health care where these devices can continuously monitor one’s health without manual interference. This constant monitoring can also help detect anomalies that may not manifest during a visit to a health care professional and can permit caregivers to remotely monitor their patients [12-14]. Several wearables have been developed for specific support in the mHealth domain and are augmented by novel solutions, such as virtual reality implemented on mobile devices [15]. Built-in sensors such as inertial measurement units (IMUs), microphones, cameras, and GPS modules can also provide insights into one’s health and have been previously used for managing conditions such as sleep apnea [16]. Bluetooth Low Energy (BLE) has been widely adopted for transferring data, and several apps have been developed that pair BLE devices with smartphones for fetching health insights. The popularity of BLE and the availability of low-cost BLE devices has opened up new avenues for continuous health monitoring in a more user-friendly manner [12]. Such sensors provide an effective platform for collecting real time metrics conveniently and less intrusively, which may be useful in medical research [17]. Recently, they have also been suggested for use in low-cost mHealth systems such as those for diagnosing pneumonia [18]. Similar suggestions have also been made for physiological measurements such as heart and respiration rates, blood oxygen saturation, and blood pressure for application in health interventions [19]. Recent studies in this area include the use of BLE devices for managing diseases ranging from asthma [20] to tissue pain and mobility issues [21]. Several studies have reviewed mHealth apps and explored them from various perspectives, such as their impact on health outcomes [22], usability [23], and even the use of integrated smartphone sensors for monitoring health conditions [16]. Despite limitations around the accuracy of the apps and peripheral such as measurement errors caused by darker skin tones and higher BMI [24] and poor energy expenditure estimations by apps [25], they remain mostly well received [26].

A study by Wisniewski et al [27] researched around the attributes of health apps where they selected 120 top-rated apps from Google and Apple app stores in different categories and evaluated them manually. Their study revealed that most apps fell under the category of *self-monitoring of health or diagnostic data by client* apps (World Health Organization classification 1.4.2) [1], indicating a higher interest in, and availability of, self-monitoring apps.

### The Use of Built-in and External Sensors

#### Overview

Most smartphones host several built-in sensors such as IMUs and GPS modules and support different wireless communication technologies such as Bluetooth and Wi-Fi. Many mHealth apps provide features such as workout tracking, medication reminders, and general health monitoring using external or built-in smartphone sensors, whereas others offer other features that may require manual data entry and include apps such as meal trackers and weight loss coaches.

#### Built-in Sensors

A recent assessment of health apps from curated health app libraries indicated that cameras were the most frequently used sensors where they were used for assessing one’s heart rate and even for automated skin cancer diagnosis [16]. Similarly, the use of microphones has been used in apps that provide respiratory therapy [28,29]. Algorithms have also been developed for processing IMU readings to monitor movement and activity levels in a noninvasive manner and are now widely used for applications in fall detection and gait analysis to track the progression of diseases such as Parkinson disease [30]. These algorithms and functions have been integrated with other data collection mechanisms described below to create complex and robust health apps, with a common example being popular fitness trackers that use external heart rate sensors along with the onboard IMUs and GPS modules.

#### BLE Standard

BLE standard was originally designed with a focus on low cost, bandwidth, power consumption, and complexity and has allowed developers to design products that are more affordable than other wireless technologies such as Wi-Fi and Zigbee [31]. BLE uses profiles to define its functionality, which can cover operation procedures such as the Generic Attribute (GATT) profile, which describes procedures for exchanging data between devices and defines data models for the same. As several implementations can be made using GATT to exchange different types of data, the Bluetooth Special Interest Group (SIG) has defined a set of use cases and specific profiles that cover the required procedures and data structures. These have been defined using GATT services and characteristics and include profiles for securely transferring health-related metrics [32] such as heart rate and blood pressure. Given that predefined profiles may not completely cover all apps, the Bluetooth SIG also permits device manufacturers to create their own vendor-specific profiles.

**Figure 1:**
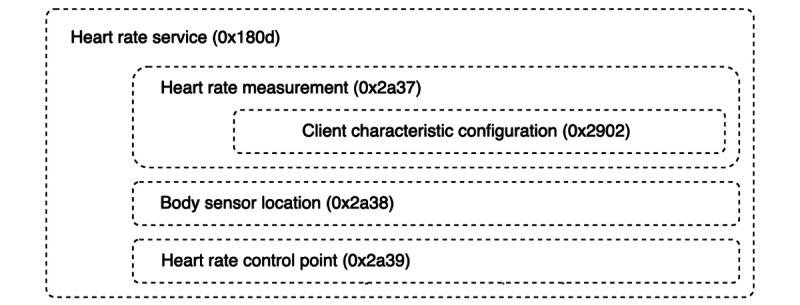
Hierarchy of Bluetooth Low Energy heart rate service with uuid16 attribute representation (adapted from Bluetooth Specification—Heart Rate Service [34]).

GATT provides a framework for data transfer and device operations, and apps based on BLE are required to comply with its specifications [31]. Data are exchanged between devices using the smallest addressable data units described by GATT—attributes. These are identified by 128-bit universally unique identifiers (UUIDs), which can also be represented using 16- (uuid16) or 32-bit (uuid32) shortened versions, with all currently SIG-assigned UUIDs being the uuid16 type [33]. The attributes are organized into nested blocks—services, which may contain 0 or more related characteristics, which, in turn, may also contain 0 or more descriptors [31]. As an example, Figure 1 describes the Bluetooth SIG-defined heart rate service specification [34].

As the GATT structure is strictly enforced for all BLE-compatible devices, any client app that intends to exchange data with them needs to either discover each exposed service or be aware of relevant services and characteristics. For specific use cases, apps would require UUID descriptions in their code for connecting with peripherals and identifying the services and subsequently reading exposed characteristics. Thus, an analysis of Android packages to extract these UUIDs would help us to identify not only those apps using peripherals to collect health metrics but also the use of standard and vendor-specific services. Apps have been previously analyzed to identify the use of BLE peripherals; however, the focus of existing works in the domain has been around security assessment and identification of vulnerabilities [35]. Tools such as BLEScope [35] and BLECryptracer [36] have been created for the same; however, they have not been used to identify the types of services supported by health apps.

### Objectives

Recent exploration of the domain has also revealed interconnectivity and convenience as 2 factors impacting user experience [37]. This is even more important today, given the thousands of health apps with overlapping features and the user tendency to use more than one app [2]. Although they are mostly data driven, there is limited work around the analysis of existing mHealth apps to identify what data are collected and how, an understanding of which can help develop better health apps and eventually improve technology adoption. Therefore, our objective is to analyze a set of free mHealth apps to investigate the use of peripherals along with built-in sensors as an indicator of the collected data and provided features.

## Methods

### Overview

**Figure 2:**
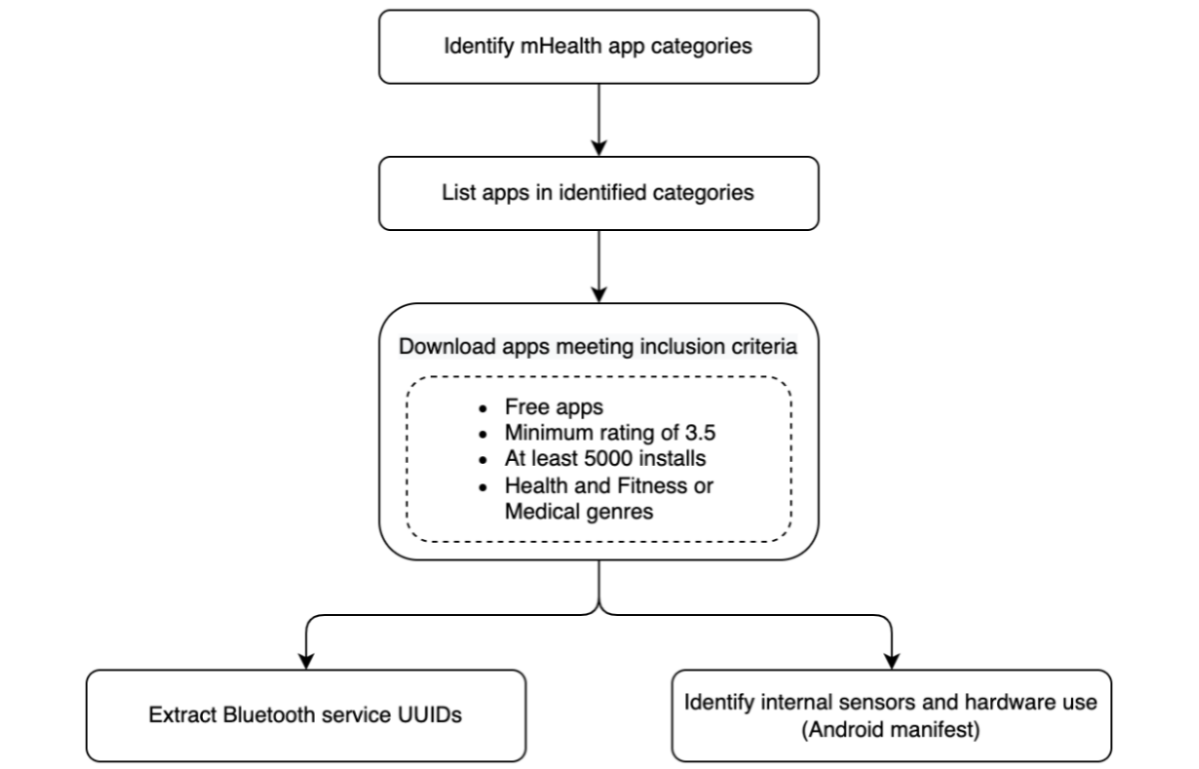
Data collection methodology. mHealth: mobile health; UUID: universally unique identifier.

The Google Play Store is the official hub for downloading Android apps and offers over 100,000 mHealth apps [38]. Given the availability of several curated health app repositories, we explored the app categories described in one major curated app list—MyHealthApps [39]. Through a search on the Play Store using terms identified from this library, we identified apps that we then downloaded and analyzed. Figure 2 shows a high-level overview of the methodology, which is discussed in detail later.

### Identifying and Collecting mHealth Apps

We referred to the categories and subcategories of mHealth apps defined by the MyHealthApps library to guide our search, and we identified 15 categories with their respective subcategories listed in Table 1. We wrote a script around a Play Store scraper [40] that returned apps from the United States with English as the default language and gave us detailed information about the apps, and the results indicated that not all apps matching the search terms were related to health but included other genres also such as *News & Magazines* and *Tools*. Although several apps of interest fell under the *Health & Fitness* or *Medical* categories, many were classified under other groups and were excluded from our list. For example, the search term *Blood Pressure* returned a set of 250 apps with 113 health and fitness apps and 89 medical apps (as of November 2020). Our selection criteria for the apps had the following four key conditions to include a large set of more popular, accessible, and quality mHealth apps: free apps, rating >3.5, number of installs >5000, and health and fitness or medical apps

However, as we also wanted to include comparatively new apps along with well-established ones, we did not consider a minimum number of ratings.

After filtering the list and removing duplicates, the remaining apps were downloaded to our test machine from a mirror site [41], following which files except those with the .apk extension were discarded. We investigated the use of built-in sensors such as accelerometers, gyroscopes, GPS modules, and even the smartphone’s camera modules in the identified apps. By analyzing app permissions, it is possible to infer to some extent what features these apps provide and how data are gathered. We were particularly interested in the use of GPS (coarse and fine locations), Bluetooth, Camera, Body Sensors, Microphones, and Activity Recognition permissions.

**Table 1:** App categories, the number of subcategories, and the total number of apps in each category (November 2020).

App category	Search terms (subcategories; n=157), n	Apps (N=38,780), n (%)
Bones and muscles	11	2745 (7.08)
Breathing and lungs	6	1493 (3.85)
Cancer	13	3237 (8.35)
Diabetes	5	1247 (3.21)
Endocrine	2	499 (1.29)
Heart, circulation, and blood	13	3238 (8.35)
HIV	1	249 (0.64)
Kidneys	1	250 (0.64)
Medication	3	749 (1.93)
Mental health	16	3991 (10.29)
Nervous system and brain	22	5469 (14.1)
Skin	3	748 (1.93)
Staying healthy	23	5744 (14.81)
Stomach, bowel, and continence	14	3493 (9.01)
Senses, mobility, and learning	24	5728 (14.77)

### Data Extraction From mHealth Apps

#### App Data Set

Our query fetched a list of 38,130 apps (as of November 2020), which were then filtered to remove duplicates in each app set and those not meeting the inclusion criteria, giving us a much smaller list of apps for analysis [42] (N=3330). Of the 3330 apps, 12 (0.36%) apps were not found on the mirror site and 67 (2.01%) returned zip files that were discarded.

#### Extracting UUIDs From Packages

To analyze the downloaded apps, we used a popular static analysis tool—AndroGuard [43], which allowed us to decompile Android packages to extract relevant details. These apps need to be aware of the relevant services, characteristics, and descriptors to connect with peripherals. However, apart from statically defined UUID strings, apps can also construct them from a base ID and a shortened version at runtime. Although tracking these IDs may be necessary to identify all the possible uses of standard services, not all apps follow this approach. Analyzing the downloaded packages with AndroGuard helped us identify the following:

- The set of permissions and hardware features requested by the apps (to help understand how data are collected by the apps)
- Apps requesting Bluetooth permission (for identifying apps that may use external peripherals)
- Statically defined UUIDs for apps using Bluetooth (for understanding the use of predefined or vendor-specified profiles)

#### The Use of Internal Sensors

As access to device hardware and other features may have security implications, Android restricts access by mandating the use of permissions declared in the app’s manifest file [44]. AndroGuard was used to identify built-in sensors accessed by mHealth apps through the declared permissions. Although the Android developer documentation recommends only using permissions necessary for the app to work as one of the best practices [45], some developers may request access to extra sensors and hardware without actually using them—a sign of a poorly developed app. However, such edge cases were not considered in this study.

#### iOS Apps

Although iOS apps also contribute to the mHealth app numbers, we limited our search to Android because of technical limitations around downloading these apps and the lack of open tools for decompiling and analyzing them. However, permission checks could be performed to indicate the types of hardware features used by these apps. We randomly selected 30 apps from the list of Android apps and searched for them on the iOS app store. Of these 30 apps, 25 (83%) were available on iOS, which were downloaded using Apple’s Configurator tool and unpacked to identify the hardware features used in the apps based on the app permissions.

Overall, in each step of the exploratory analysis, custom tools were built and used to automate app downloads, static analysis, data manipulation, and management. Data were then manually checked to ensure accuracy.

## Results

### The Use of Internal Sensors

**Figure 3:**
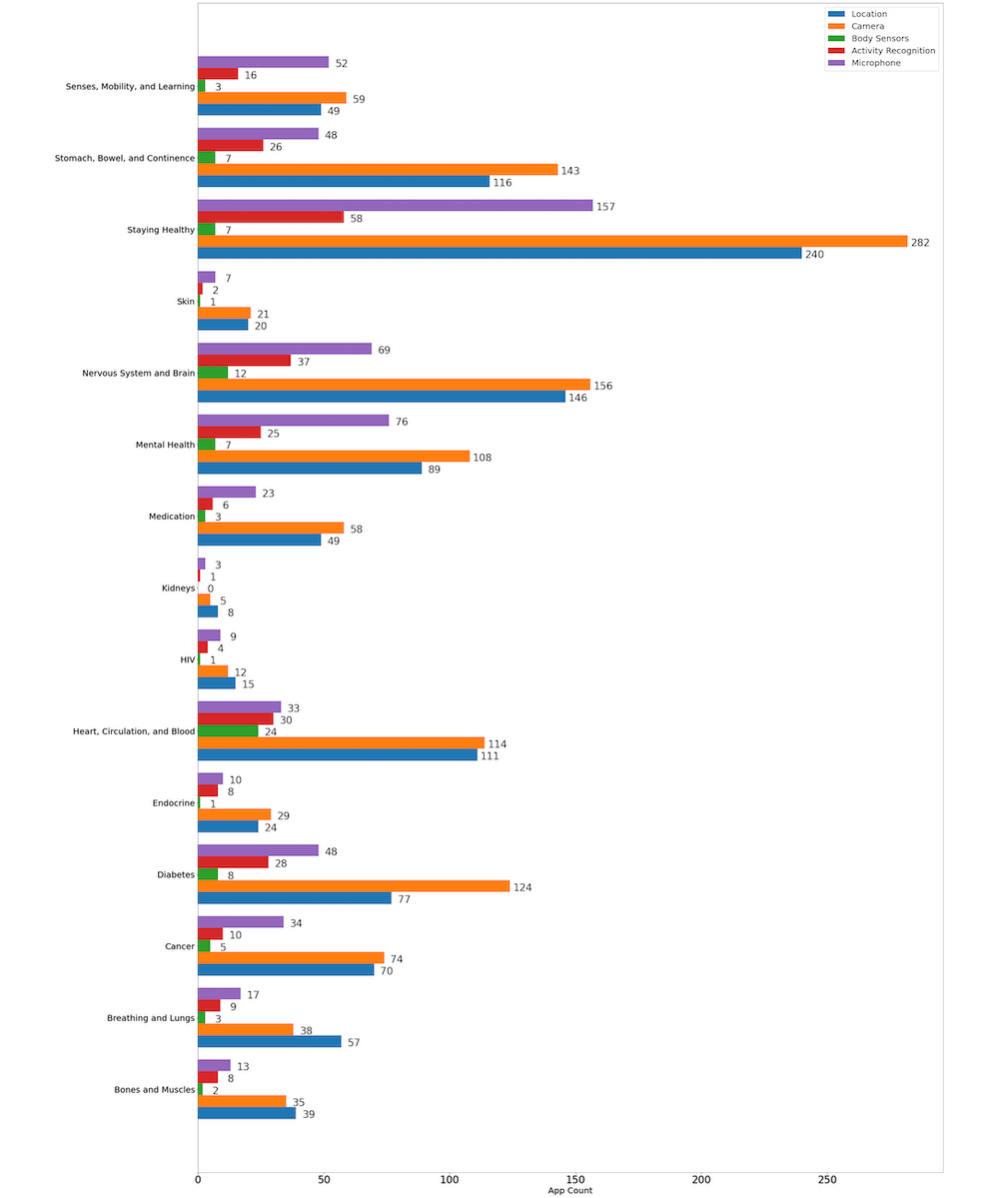
Built-in sensors used in apps across different categories.

From the analyzed set of 3251 apps, we found several apps using the coarse (*ACCESS_COARSE_LOCATION*) and fine (*ACCESS_FINE_LOCATION*) locations, suggesting the use of distance tracking as a possible feature. Similarly, several instances of activity recognition for tracking step counts (*ACTIVITY_RECOGNITION*) and a few for body sensors (*BODY_SENSORS*) were found. Smartphone cameras have also been widely used, as indicated by the presence of over 600 apps that requested the appropriate permission (*CAMERA*). Table 2 lists the number of apps using these permissions. Figure 3 shows the use of different sensors in each subset. We found that the camera being more popular across most search categories with GPS following closely, with the highest use seen in the *Staying Healthy* category. The high use of cameras is consistent with previous app reviews [16] and is discussed in the next section.

**Table 2:** Apps and requested permissions (N=3251).

Permissions (simplified)	Apps, n (%)
Coarse location	557 (17.13)
Fine location	598 (18.39)
Camera	669 (20.57)
Body sensors	36 (1.11)
Activity recognition	123 (3.78)
Audio recording	340 (10.45)

### The Use of Bluetooth Peripherals

Apps need to *know* the UUIDs of the services exposed by BLE peripherals to communicate with them and transfer data, and we found that 10.74% (349/3251) of the apps requested Bluetooth access. Table 3 lists the percentage of apps in each search category using Bluetooth. The Bluetooth SIG permits the use of vendor-specific UUIDs for different use cases, and 50.9% (259/509) of the discovered UUIDs were known and include service, characteristic, and descriptor identifiers. The unknown IDs include vendor-specific UUIDs along with those not related to Bluetooth operations; as these are not available publicly, further separation of this set was not possible.

We mapped the known UUIDs to the apps that used them, which allowed us to identify the most commonly used UUIDs and therefore, services. *Client Characteristic Configuration (00002902-0000-1000-8000-00805f9b34fb)* was found to be the most common, with 116 apps using the same UUID. However, not many standard health-related services were identified, and we note that only the Heart Rate Measurement (41/3251, 1.26%) and the Heart Rate Service (40/3251, 1.23%) UUIDs were found in the top 10, with 1.2% (30/3251) of the analyzed apps using these services (Table 4).

We analyzed the permissions requested in the 25 iOS app versions of the selected Android apps. Given that the apps provided the same features, the permissions were not expected to differ and were mostly identical in the main categories of interest—Bluetooth, Camera, Microphone, Activity Recognition, and Location (Multimedia Appendix 1). A few minor deviations were observed on both platforms where some permissions did not match (eg, 2 Android variants requested near-field communication, which was not available on iOS). We could not analyze the BLE UUIDs in the apps because of the lack of open tools such as AndroGuard on iOS. However, given that both iOS and Android versions of an app are connected to similar hardware, the UUIDs are expected to be the same for the same group of apps.

**Table 3:** Percentage of apps in each category using Bluetooth.

App category	Percentage of apps using Bluetooth, n/N (%)
Bones and muscles	16/216 (7.4)
Breathing and lungs	29/188 (15.4)
Cancer	23/326 (7.1)
Diabetes	59/374 (15.8)
Endocrine	12/91 (13.2)
Heart, circulation, and blood	72/495 (14.5)
HIV	14/71 (19.7)
Kidneys	4/67 (6)
Medication	16/258 (6.2)
Mental health	53/505 (10.5)
Nervous system and brain	80/649 (12.3)
Skin	4/67 (6)
Staying healthy	145/1340 (10.8)
Stomach, bowel, and continence	58/454 (12.8)
Senses, mobility, and learning	49/391 (12.5)

**Table 4:** Services and the number of apps using them (N=3251).

Generic Attribute service	Apps, n (%)
Heart rate measurement	41 (1.26)
Glucose measurement	26 (0.79)
Running speed and cadence	14 (0.43)
Cycling speed and cadence	13 (0.39)
Blood-pressure measurement	13 (0.39)
Body composition measurement	12 (0.36)
Weight measurement	10 (0.3)

## Discussion

### Overview

End users tend to deal with multiple mHealth apps to manage their health and well-being, with even health care providers referring to more than one app as one may not provide all the details they need [2]. These apps do not share a consistent user interface, sensors, or a common mHealth data model, leading to poor overall user experience. To address this problem, there is a need for a comprehensive mHealth data collection model and catalog of sensors to develop robust app development guidelines and frameworks. This study represents the first step in this road map. We reviewed data collection in 3251 mHealth apps to understand what health data are collected and how apps collect them with a focus on built-in and external Bluetooth-based sensors.

### Data Collection

Our findings indicate that although there is an increasing use of smart wearables and the increasing popularity of peripherals, not many apps use them to collect health data. Similarly, not many apps were found to use built-in smartphone sensors. Our results are consistent with a recent study by Wisniewski et al [27], where most apps relied on manual entry with limited support for any wearables. Their reliance on manual reviews limited their study to 120 apps for mental health, which we were able to extend by automating the app review process. However, one drawback of our approach is that we cannot conclusively determine where the data were being used or what they were used for.

Our results show 20.57% (669/3251) and 18.39% (598/3251) of the apps used the camera and GPS modules, respectively, which are also the most used sensors across each app category, with the highest occurrence in the *Staying Healthy* set. This was expected as this category includes apps around diet and exercise where images and location data may be used for tracking meals and physical outdoor activities. We also expected a similar trend favoring cameras in the *Heart, Circulation and Blood* category. We were also surprised by the relatively high use of location and images in *Nervous System and Brain* apps, which may indicate the increasing acceptance of these data types in different apps. This can be an indication of useful features, such as scanning an item (eg, medication) or tracking movement. However, it may also indicate poor app design where access to sensors is requested without actually using them. Unsurprisingly, the lowest occurrence of these sensors was found in more medical apps as opposed to health and fitness apps, where categories such as HIV and Kidneys may not have any use of currently available built-in sensors at all. Apps were also found to use the microphone with the highest occurrence in the *Staying Healthy* category where its use can range from call features to speech analysis to tracking one’s sleep.

Given the popularity of health wearables and peripherals, we expected to find a significant number of apps supporting them for passive data collection. However, our results indicated the opposite. We found 10.74% (349/3251) of the apps requesting Bluetooth access, of which only about half of the discovered UUIDs were found to be the standard Bluetooth services with the remaining unknown. Apart from unrelated IDs, this also indicates that most devices and apps used proprietary algorithms, limiting their compatibility and use [46]. However, of those that were known, very few were related to health, with the highest occurrence being the Heart Rate Measurement service in 40 apps (1.2%). Vendor-specific IDs (almost 50% of the reported apps using Bluetooth, n=250, 49.1%) may be used for any purpose, as defined by device manufacturers, making it difficult to identify the data transferred through those services. Besides the possibility of the UUIDs not being detected, this suggests that despite the growing popularity of wearables, they are restricted to a few manufacturers with limited apps using proprietary services and formats.

As we rejected apps with low ratings and downloads, we may also have skipped several bespoke apps used for specific cases or by small groups. These can include apps developed for research studies and specialized devices that may not be widely available. Similarly, as Google restricts search results to 250 items per search term, we were also limited in our app search. The analyzed data also indicated the presence of other known health-related services in a smaller number and showed the use of Heart Rate, Glucose, and Body Composition as the more common services provided by peripheral devices.

### Data Sharing

mHealth devices and apps have been found to be useful for collecting clinical insights [47], which shows their potential not only in personal use but also in clinical apps where integration with electronic health records can help improve health outcomes. Newer apps integrate with frameworks such as Apple Health or Google Fit that allow data aggregation and sharing; however, they also require installation of more than one app—a challenge that deters end users. Here, a platform integrating a diverse set of apps, health records, and sensors could improve this aspect of mHealth apps with functionality and usability blending in seamlessly, potentially improving health outcomes.

### Tools and Data Set

In addition to app analysis, our contribution also includes the raw data set, including collected app details along with the extracted data comprising app permissions and identified UUIDs. Our tool for downloading and analyzing apps is also included in our repository, which is available on GitHub [48], and would be beneficial for future studies related to mHealth app analysis.

Overall, our results suggest a more common use of manual entry (where automated data collection is possible), which, apart from being less reliable, also degrades user experience, leading to more users abandoning health apps [49]. Although usability is subjective, limited support for passive data collection with internal and external sensors can have a negative impact on app experience, which can lead to reduced adoption by end users—sidestepping any benefits the apps could offer. Therefore, it is critical to understand the importance of peripherals and built-in sensors in modern health solutions and integrating them in a clinically acceptable manner with health apps.

However, the main limitation of our work arises from automated data extraction, where we could not capture more nuanced details such as where the data from these sensors are being used and requires further investigation. Many valid health apps such as reference apps, management apps (weight and diet), and calculators (body composition, drug dosage, etc) may also be classified under other categories such as *Education* and *Books & Reference* and were rejected. Similarly, because of the difference in app numbers in each category, a comparison between them may be biased.

Given the presence of over 300,000 apps in app stores, analyzing all of them was not feasible and using a curated app list was a better approach for identifying different apps as they would be closer to the domain than manually searching through thousands of apps. As more health apps are widely available today for managing one’s health, we believe that our results are relevant where accuracy of health data and wider integration would be important for better health care delivery. Although app analysis can be performed manually, we chose to automate the process, which ignored possible data sources such as developer descriptions and user reviews. Similarly, our results are based on limited app categories where the use of sensors and wearables may not be feasible (eg, *Medication* or *Mental Health*), and we acknowledge that these results may not be generalizable to the entire domain. Therefore, there is also a need to explore new apps of current sensors in these areas to improve data collection.

Given the potential of mHealth apps to improve an end user’s health, adherence to regular use is essential, which can only be ensured if such apps are intuitive and convenient to use. In the larger context of a connected, Internet of thing–enabled ecosystem, apps would play an important role as an interface. This indicates a need to integrate more peripherals with health apps to collect user data, which, along with built-in sensors, could ultimately help improve health outcomes. To that end, we envision a connected ecosystem of mini health apps, sensors, and health records as a key mHealth technology of the future. We plan to use these results to develop a single mHealth platform for aggregating several wearables and health apps as mini health services, which we believe would provide a much better experience to end users. We have built a prototype of such a platform with health micro-mHealth apps [50], an introduction to which is planned in our upcoming work followed by a study to understand its impact on user experience and technology adoption.

### Conclusions

Given that user studies on app experience have highlighted convenience and data interconnectivity and aggregation as important factors, automating data collection can improve user experience, especially in apps requiring access to health metrics. However, a limited number of apps in our search were found to do so, indicating the need for more focus on integrating more peripherals and built-in sensors for health apps.

Our analysis of 3251 apps indicates that <10.74% (n=349) of the apps use smart devices and wearables to gather health metrics from users. In this set, extracted UUIDs show that very few apps used standard health-related Bluetooth services, with the most popular service being Heart Rate Measurement. Several apps have been found to use custom services that affect the interoperability of devices with different apps. Here, using standard profiles may be beneficial, as more apps would be able to interact with these devices, giving end users more options. Similarly, several apps were found to request access to device hardware features, such as GPS and camera, indicating the increasing acceptance of these devices. However, their numbers remain small, indicating the need for more research into using them in health apps.

Although manual entry may be inevitable for some apps, a significant number of apps requiring manual data entry were found in our set, highlighting the need to focus more on developing mHealth apps that automate health data collection. As several apps for research and health studies have been published, a better approach for developing and consuming mHealth apps is required. Overall, our findings can guide the design of future mHealth apps and has a positive impact on improving mHealth data collection in these apps.

## Appendix

Multimedia Appendix 1 Android and iOS permission comparison.

## Abbreviations

BLE: Bluetooth low energy
GATT: Generic Attribute
IMU: inertial measurement unit
mHealth: mobile health
SIG: Special Interest Group
UUID: universally unique identifier
